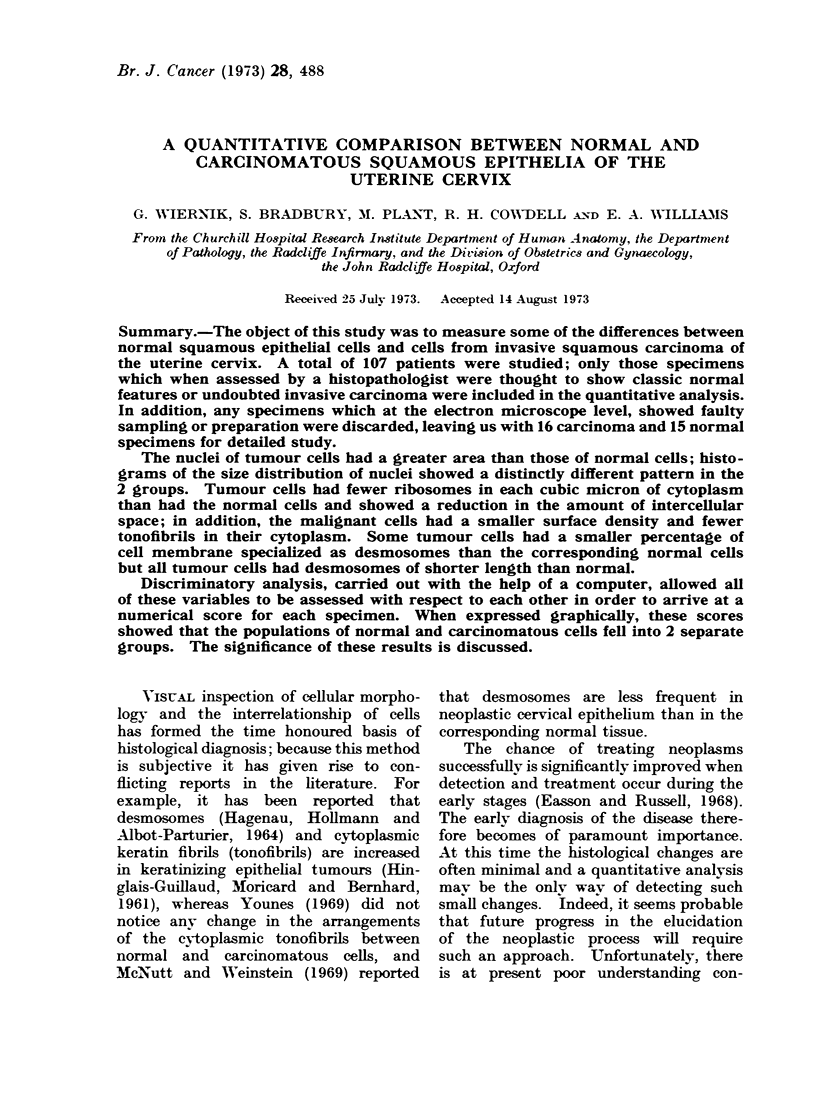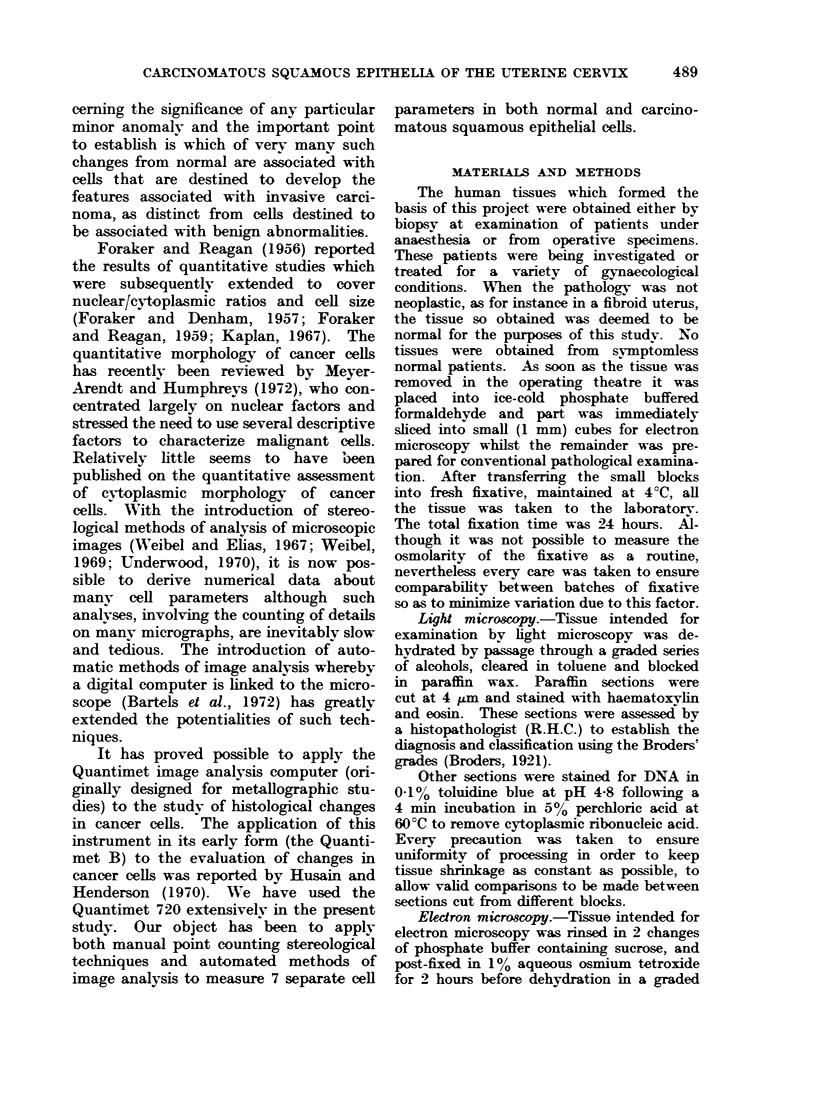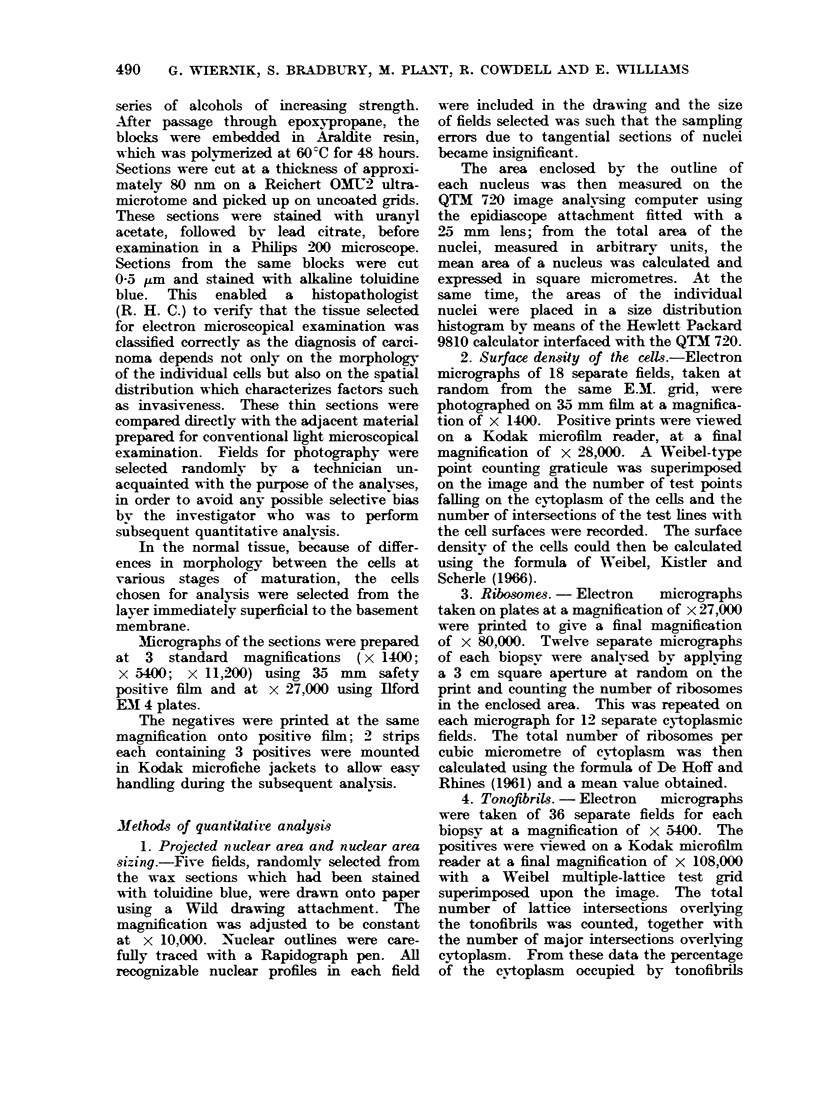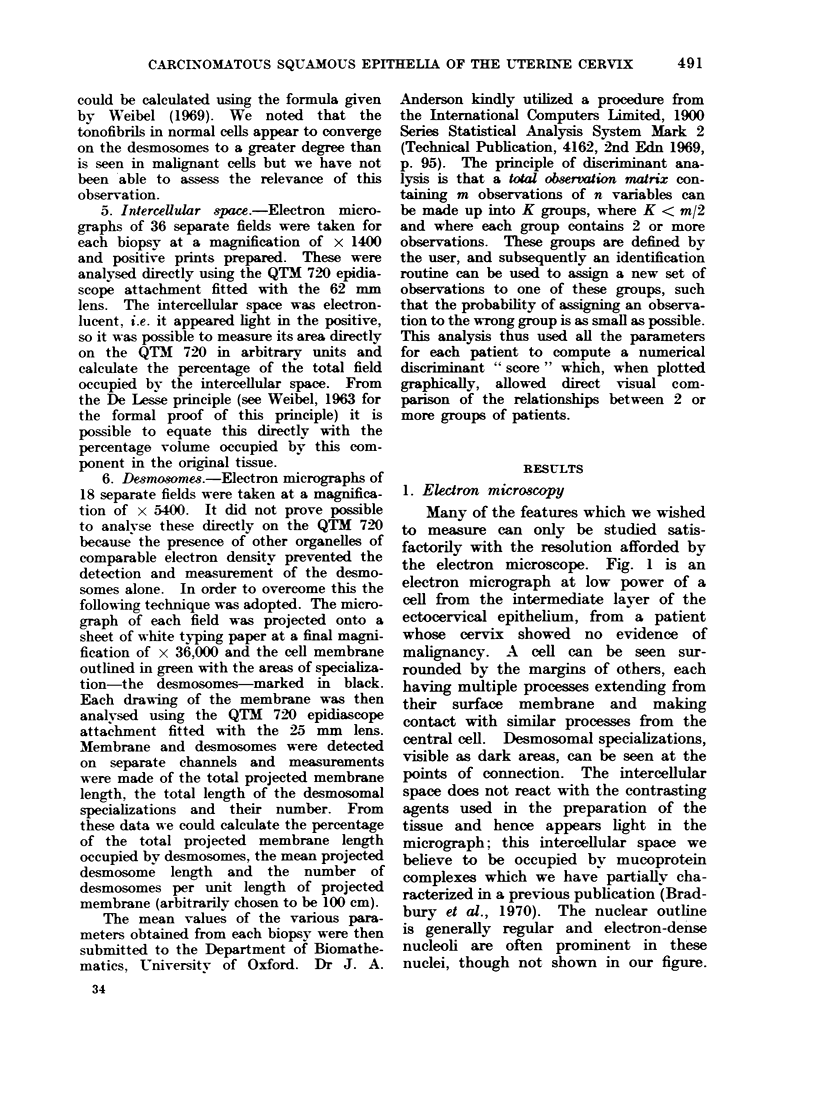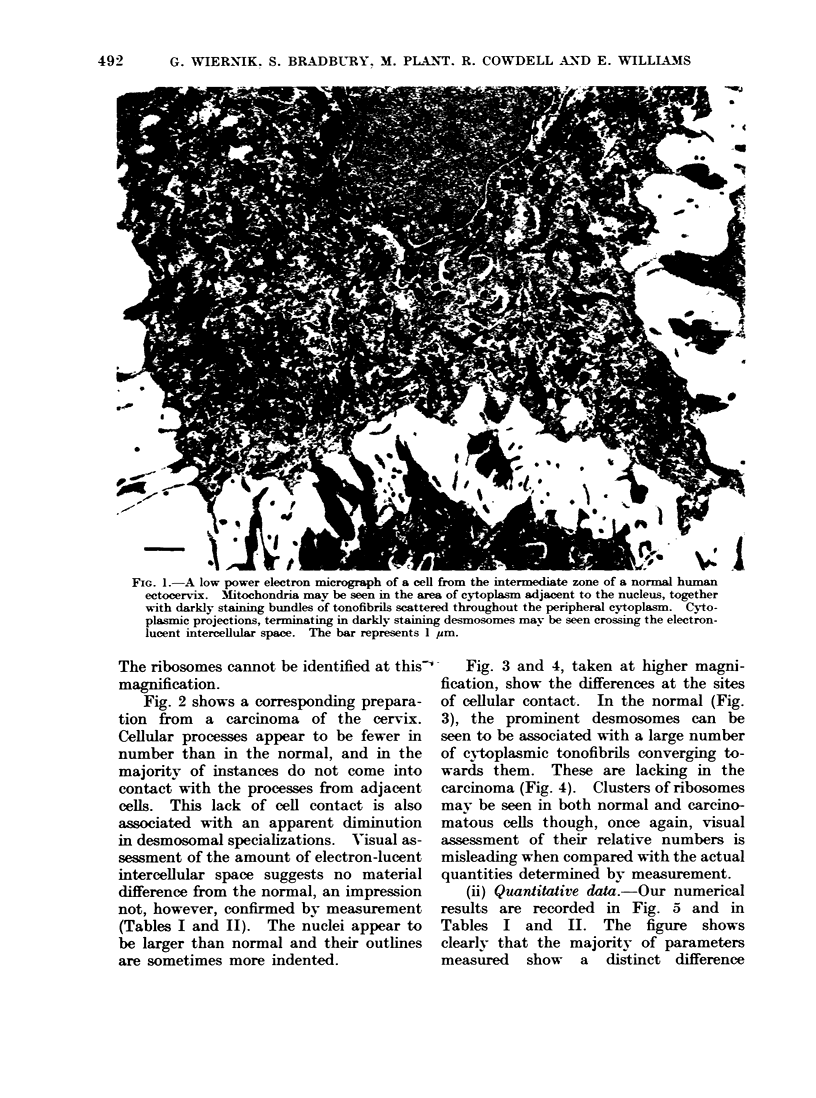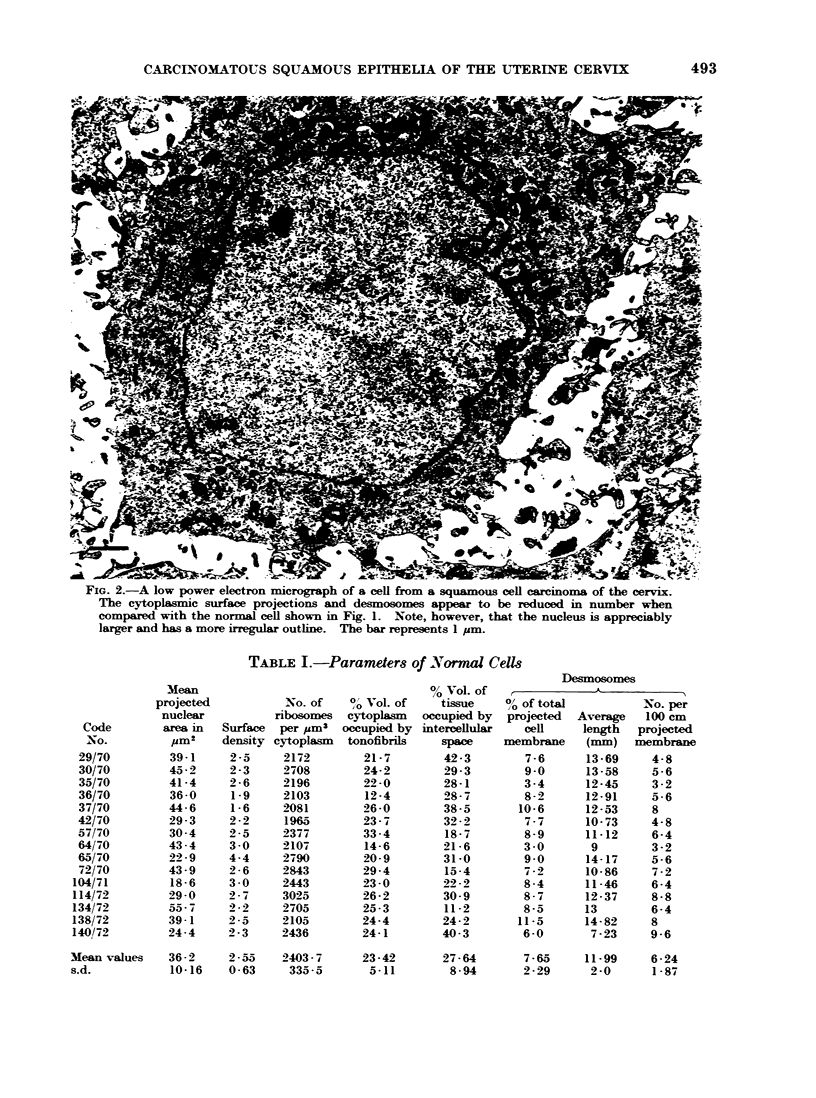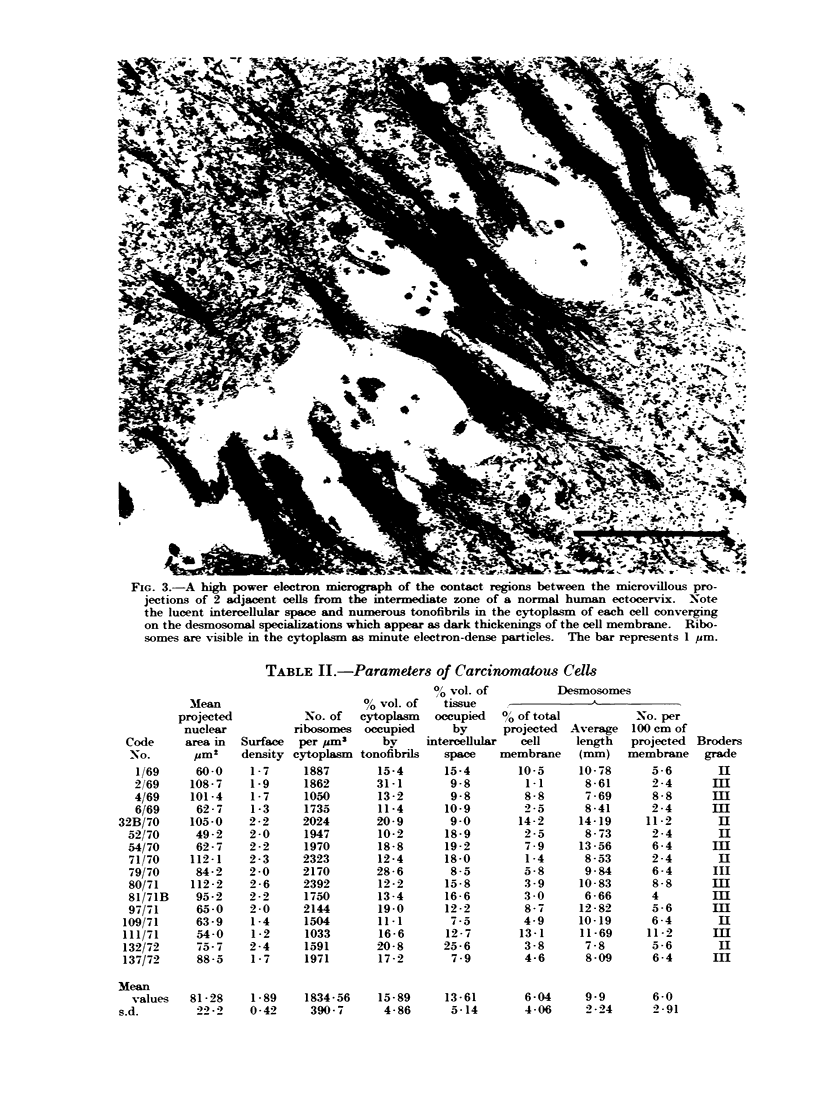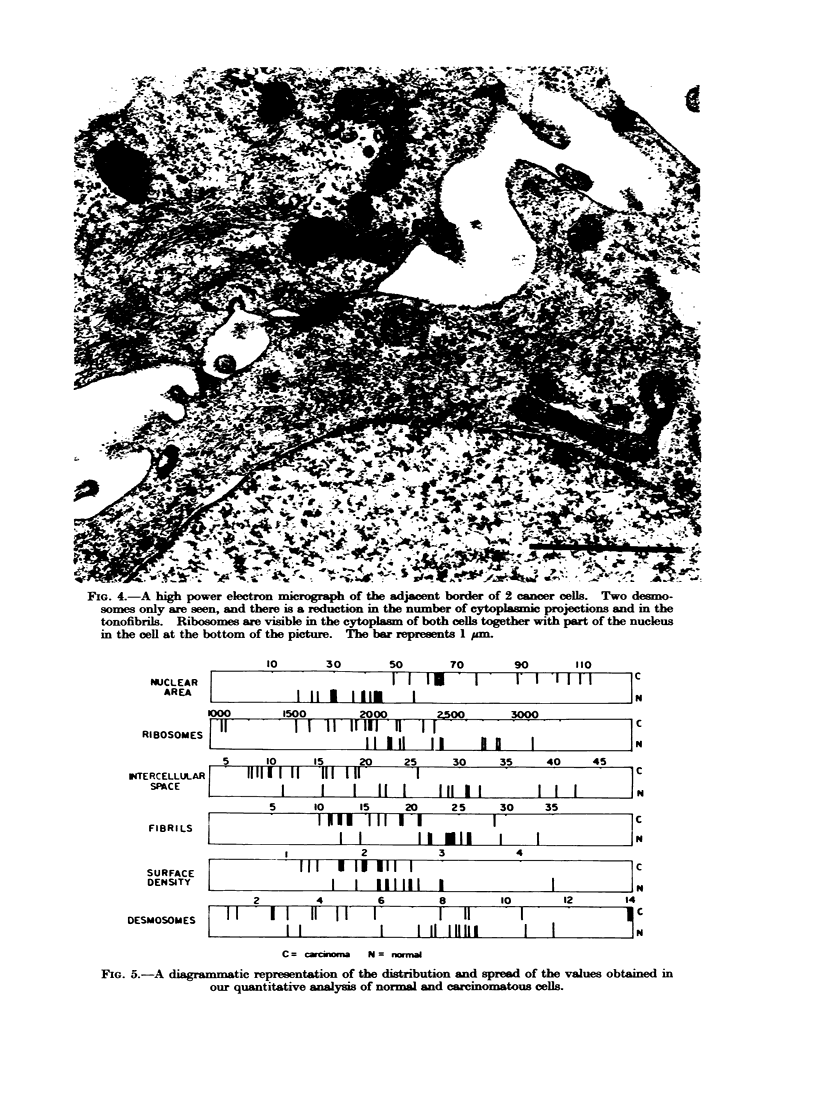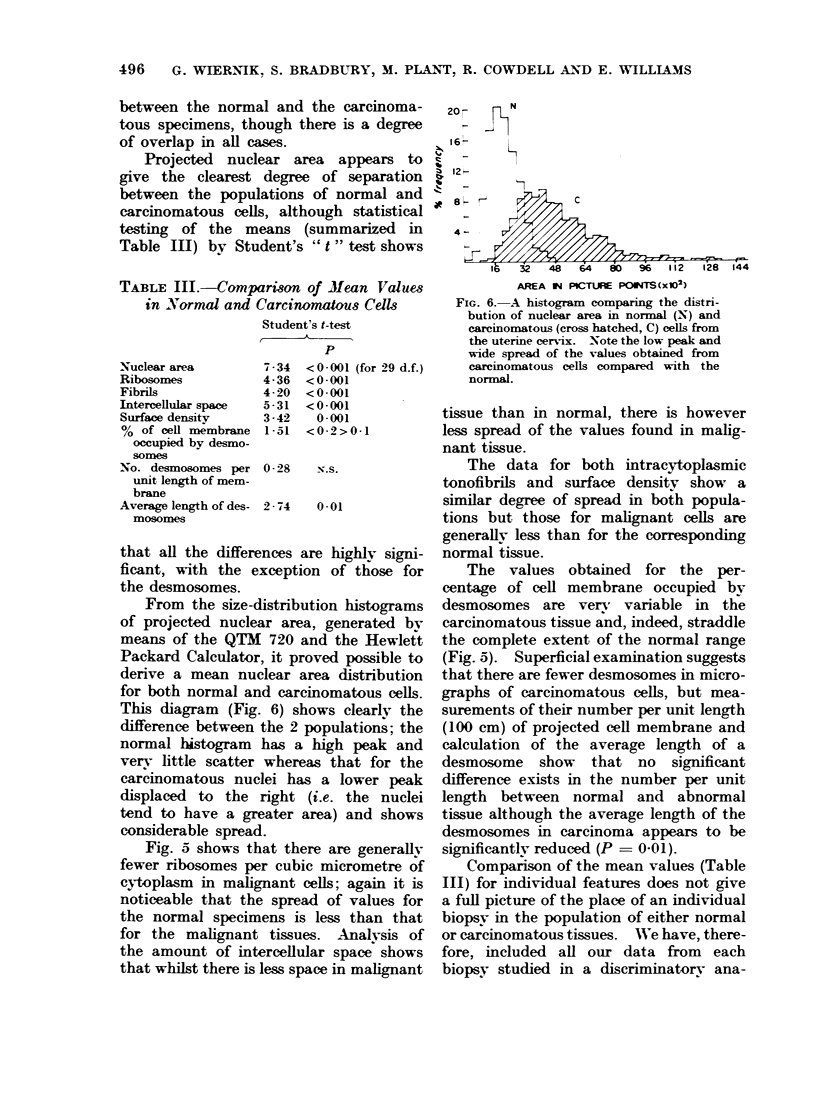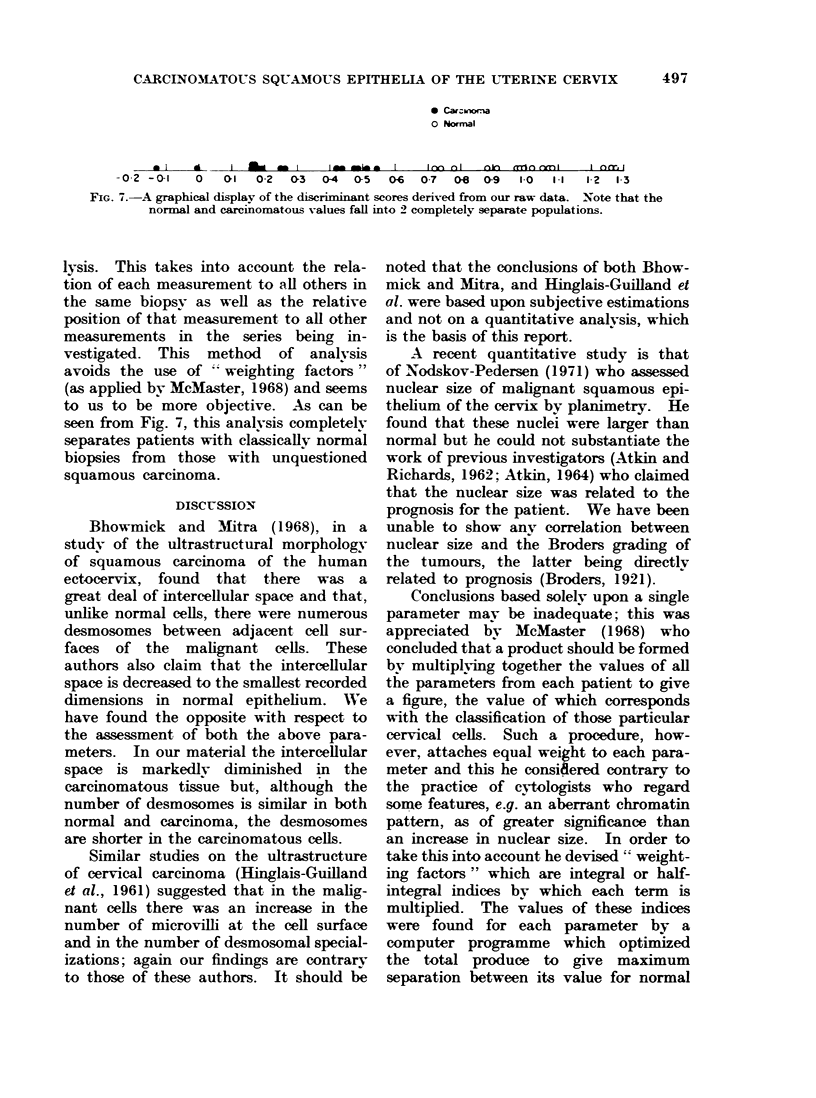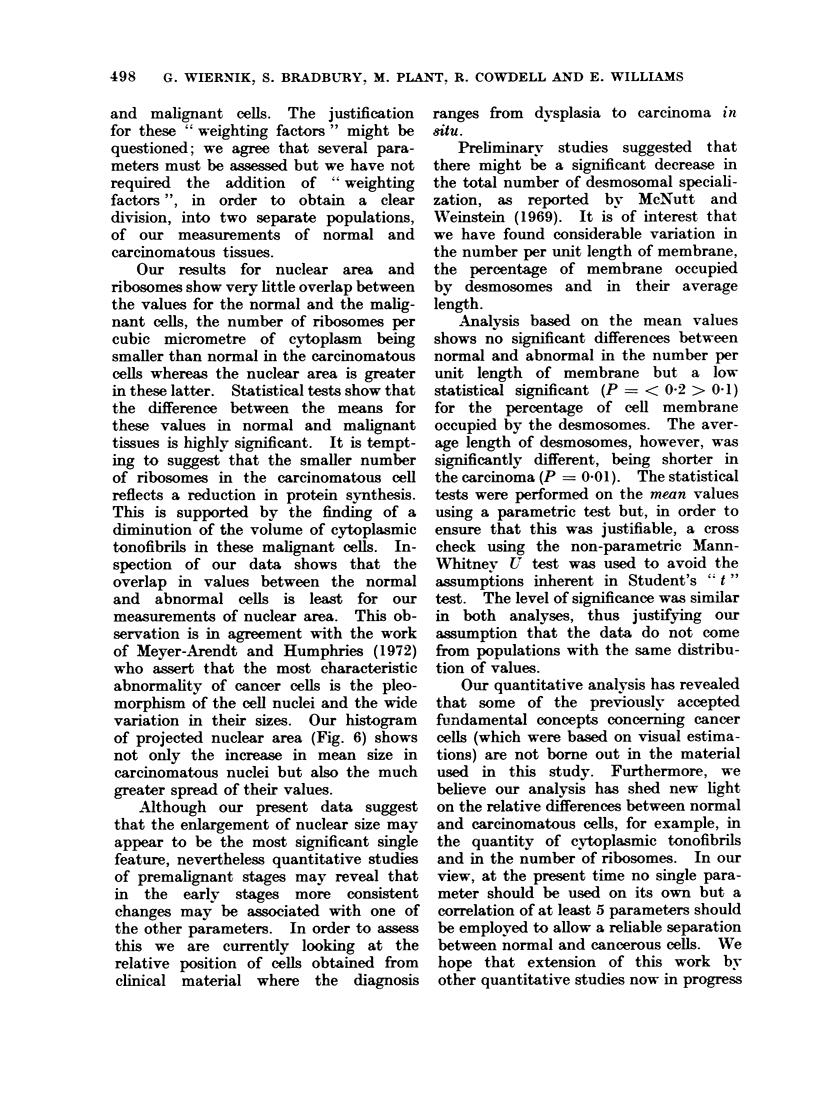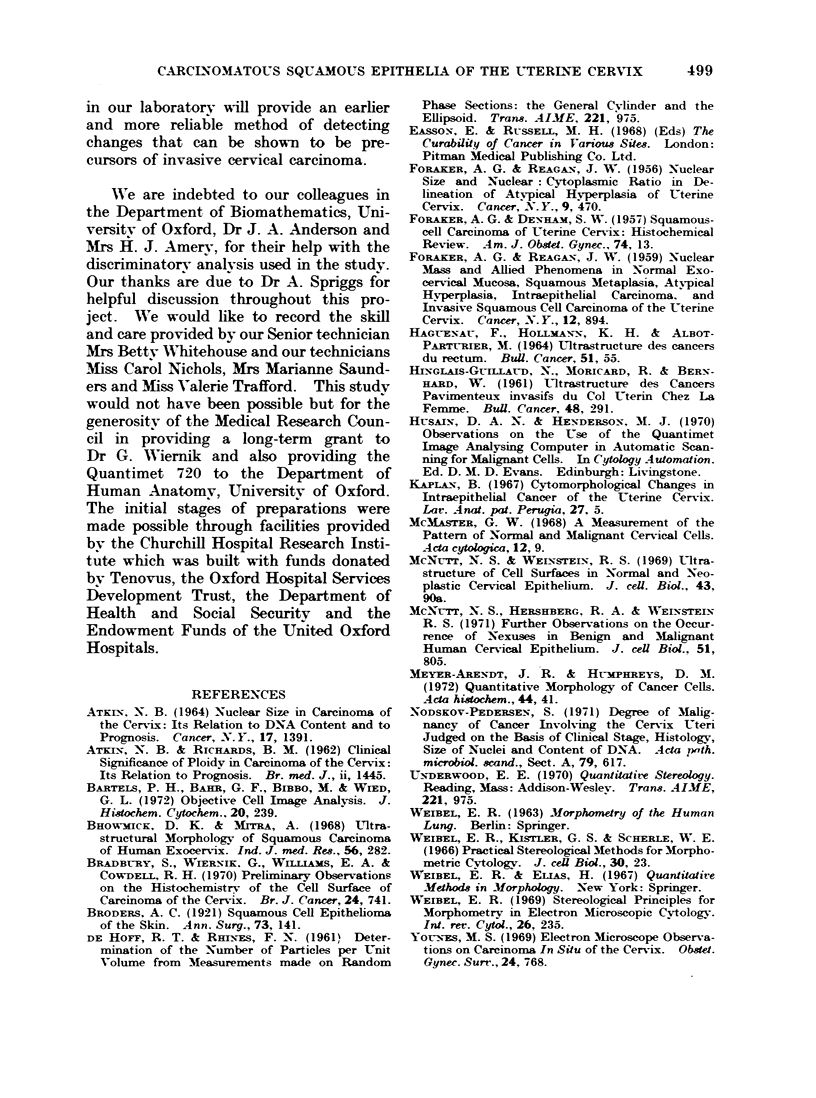# A Quantitative Comparison Between Normal and Carcinomatous Squamous Epithelia of the Uterine Cervix

**DOI:** 10.1038/bjc.1973.178

**Published:** 1973-12

**Authors:** G. Wiernik, S. Bradbury, M. Plant, R. H. Cowdell, E. A. Williams

## Abstract

**Images:**


*Br. J. Cancer* (1973) **28**, 488

### A QUANTITATIVE COMPARISON BETWEEN NORMAL AND CARCINOMATOUS SQUAMOUS EPITHELIA OF THE UTERINE CERVIX

G. WIERNIK, S. BRADBURY, M. PLANT, R. H. COWDELL AND E. A. WILLIAMS

*From the Churchill Hospital Research Institute Department of Human Anatomy, the Department of Pathology, the Radcliffe Infirmary, and the Division of Obstetrics and Gynaecology, the John Radcliffe Hospital, Oxford*

Received 25 July 1973. Accepted 14 August 1973

**Summary.—The object of this study was to measure some of the differences between normal squamous epithelial cells and cells from invasive squamous carcinoma of the uterine cervix. A total of 107 patients were studied; only those specimens which when assessed by a histopathologist were thought to show classic normal features or undoubted invasive carcinoma were included in the quantitative analysis. In addition, any specimens which at the electron microscope level, showed faulty sampling or preparation were discarded, leaving us with 16 carcinoma and 15 normal specimens for detailed study.**

**The nuclei of tumour cells had a greater area than those of normal cells; histograms of the size distribution of nuclei showed a distinctly different pattern in the 2 groups. Tumour cells had fewer ribosomes in each cubic micron of cytoplasm than had the normal cells and showed a reduction in the amount of intercellular space; in addition, the malignant cells had a smaller surface density and fewer tonofibrils in their cytoplasm. Some tumour cells had a smaller percentage of cell membrane specialized as desmosomes than the corresponding normal cells but all tumour cells had desmosomes of shorter length than normal.**

**Discriminatory analysis, carried out with the help of a computer, allowed all of these variables to be assessed with respect to each other in order to arrive at a numerical score for each specimen. When expressed graphically, these scores showed that the populations of normal and carcinomatous cells fell into 2 separate groups. The significance of these results is discussed.**

VISUAL inspection of cellular morphology and the interrelationship of cells has formed the time honoured basis of histological diagnosis; because this method is subjective it has given rise to conflicting reports in the literature. For example, it has been reported that desmosomes (Hagenau, Hollmann and Albot-Parturier, 1964) and cytoplasmic keratin fibrils (tonofibrils) are increased in keratinizing epithelial tumours (Hinglais-Guillaud, Moricard and Bernhard, 1961), whereas Younes (1969) did not notice any change in the arrangements of the cytoplasmic tonofibrils between normal and carcinomatous cells, and McNutt and Weinstein (1969) reported that desmosomes are less frequent in neoplastic cervical epithelium than in the corresponding normal tissue.

The chance of treating neoplasms successfully is significantly improved when detection and treatment occur during the early stages (Easson and Russell, 1968). The early diagnosis of the disease therefore becomes of paramount importance. At this time the histological changes are often minimal and a quantitative analysis may be the only way of detecting such small changes. Indeed, it seems probable that future progress in the elucidation of the neoplastic process will require such an approach. Unfortunately, there is at present poor understanding concerning the significance of any particular minor anomaly and the important point to establish is which of very many such changes from normal are associated with cells that are destined to develop the features associated with invasive carcinoma, as distinct from cells destined to be associated with benign abnormalities.

Foraker and Reagan (1956) reported the results of quantitative studies which were subsequently extended to cover nuclear/cytoplasmic ratios and cell size (Foraker and Denham, 1957; Foraker and Reagan, 1959; Kaplan, 1967). The quantitative morphology of cancer cells has recently been reviewed by Meyer-Arendt and Humphreys (1972), who concentrated largely on nuclear factors and stressed the need to use several descriptive factors to characterize malignant cells. Relatively little seems to have been published on the quantitative assessment of cytoplasmic morphology of cancer cells. With the introduction of stereological methods of analysis of microscopic images (Weibel and Elias, 1967; Weibel, 1969; Underwood, 1970), it is now possible to derive numerical data about many cell parameters although such analyses, involving the counting of details on many micrographs, are inevitably slow and tedious. The introduction of automatic methods of image analysis whereby a digital computer is linked to the microscope (Bartels *et al.*, 1972) has greatly extended the potentialities of such techniques.

It has proved possible to apply the Quantimet image analysis computer (originally designed for metallographic studies) to the study of histological changes in cancer cells. The application of this instrument in its early form (the Quantimet B) to the evaluation of changes in cancer cells was reported by Husain and Henderson (1970). We have used the Quantimet 720 extensively in the present study. Our object has been to apply both manual point counting stereological techniques and automated methods of image analysis to measure 7 separate cell parameters in both normal and carcinomatous squamous epithelial cells.

### MATERIALS AND METHODS

The human tissues which formed the basis of this project were obtained either by biopsy at examination of patients under anaesthesia or from operative specimens. These patients were being investigated or treated for a variety of gynaecological conditions. When the pathology was not neoplastic, as for instance in a fibroid uterus, the tissue so obtained was deemed to be normal for the purposes of this study. No tissues were obtained from symptomless normal patients. As soon as the tissue was removed in the operating theatre it was placed into ice-cold phosphate buffered formaldehyde and part was immediately sliced into small (1 mm) cubes for electron microscopy whilst the remainder was prepared for conventional pathological examination. After transferring the small blocks into fresh fixative, maintained at 4°C, all the tissue was taken to the laboratory. The total fixation time was 24 hours. Although it was not possible to measure the osmolarity of the fixative as a routine, nevertheless every care was taken to ensure comparability between batches of fixative so as to minimize variation due to this factor.

*Light microscopy.*—Tissue intended for examination by light microscopy was dehydrated by passage through a graded series of alcohols, cleared in toluene and blocked in paraffin wax. Paraffin sections were cut at 4 $\mu$m and stained with haematoxylin and eosin. These sections were assessed by a histopathologist (R.H.C.) to establish the diagnosis and classification using the Broders' grades (Broders, 1921).

Other sections were stained for DNA in 0·1% toluidine blue at pH 4·8 following a 4 min incubation in 5% perchloric acid at 60°C to remove cytoplasmic ribonucleic acid. Every precaution was taken to ensure uniformity of processing in order to keep tissue shrinkage as constant as possible, to allow valid comparisons to be made between sections cut from different blocks.

*Electron microscopy.*—Tissue intended for electron microscopy was rinsed in 2 changes of phosphate buffer containing sucrose, and post-fixed in 1% aqueous osmium tetroxide for 2 hours before dehydration in a graded series of alcohols of increasing strength. After passage through epoxypropane, the blocks were embedded in Araldite resin, which was polymerized at 60°C for 48 hours. Sections were cut at a thickness of approximately 80 nm on a Reichert OMU2 ultramicrotome and picked up on uncoated grids. These sections were stained with uranyl acetate, followed by lead citrate, before examination in a Philips 200 microscope. Sections from the same blocks were cut 0·5 $\mu$m and stained with alkaline toluidine blue. This enabled a histopathologist (R. H. C.) to verify that the tissue selected for electron microscopical examination was classified correctly as the diagnosis of carcinoma depends not only on the morphology of the individual cells but also on the spatial distribution which characterizes factors such as invasiveness. These thin sections were compared directly with the adjacent material prepared for conventional light microscopical examination. Fields for photography were selected randomly by a technician unacquainted with the purpose of the analyses, in order to avoid any possible selective bias by the investigator who was to perform subsequent quantitative analysis.

In the normal tissue, because of differences in morphology between the cells at various stages of maturation, the cells chosen for analysis were selected from the layer immediately superficial to the basement membrane.

Micrographs of the sections were prepared at 3 standard magnifications (× 1400; × 5400; × 11,200) using 35 mm safety positive film and at × 27,000 using Ilford EM 4 plates.

The negatives were printed at the same magnification onto positive film; 2 strips each containing 3 positives were mounted in Kodak microfiche jackets to allow easy handling during the subsequent analysis.

### *Methods of quantitative analysis*

1. *Projected nuclear area and nuclear area sizing.*—Five fields, randomly selected from the wax sections which had been stained with toluidine blue, were drawn onto paper using a Wild drawing attachment. The magnification was adjusted to be constant at × 10,000. Nuclear outlines were carefully traced with a Rapidograph pen. All recognizable nuclear profiles in each field were included in the drawing and the size of fields selected was such that the sampling errors due to tangential sections of nuclei became insignificant.

The area enclosed by the outline of each nucleus was then measured on the QTM 720 image analysing computer using the epidiascope attachment fitted with a 25 mm lens; from the total area of the nuclei, measured in arbitrary units, the mean area of a nucleus was calculated and expressed in square micrometres. At the same time, the areas of the individual nuclei were placed in a size distribution histogram by means of the Hewlett Packard 9810 calculator interfaced with the QTM 720.

2. *Surface density of the cells.*—Electron micrographs of 18 separate fields, taken at random from the same E.M. grid, were photographed on 35 mm film at a magnification of × 1400. Positive prints were viewed on a Kodak microfilm reader, at a final magnification of × 28,000. A Weibel-type point counting graticule was superimposed on the image and the number of test points falling on the cytoplasm of the cells and the number of intersections of the test lines with the cell surfaces were recorded. The surface density of the cells could then be calculated using the formula of Weibel, Kistler and Scherle (1966).

3. *Ribosomes.* — Electron micrographs taken on plates at a magnification of ×27,000 were printed to give a final magnification of × 80,000. Twelve separate micrographs of each biopsy were analysed by applying a 3 cm square aperture at random on the print and counting the number of ribosomes in the enclosed area. This was repeated on each micrograph for 12 separate cytoplasmic fields. The total number of ribosomes per cubic micrometre of cytoplasm was then calculated using the formula of De Hoff and Rhines (1961) and a mean value obtained.

4. *Tonofibrils.* — Electron micrographs were taken of 36 separate fields for each biopsy at a magnification of × 5400. The positives were viewed on a Kodak microfilm reader at a final magnification of × 108,000 with a Weibel multiple-lattice test grid superimposed upon the image. The total number of lattice intersections overlying the tonofibrils was counted, together with the number of major intersections overlying cytoplasm. From these data the percentage of the cytoplasm occupied by tonofibrils could be calculated using the formula given by Weibel (1969). We noted that the tonofibrils in normal cells appear to converge on the desmosomes to a greater degree than is seen in malignant cells but we have not been able to assess the relevance of this observation.

5. *Intercellular space*.—Electron micrographs of 36 separate fields were taken for each biopsy at a magnification of × 1400 and positive prints prepared. These were analysed directly using the QTM 720 epidiascope attachment fitted with the 62 mm lens. The intercellular space was electron-lucent, *i.e.* it appeared light in the positive, so it was possible to measure its area directly on the QTM 720 in arbitrary units and calculate the percentage of the total field occupied by the intercellular space. From the De Lesse principle (see Weibel, 1963 for the formal proof of this principle) it is possible to equate this directly with the percentage volume occupied by this component in the original tissue.

6. *Desmosomes*.—Electron micrographs of 18 separate fields were taken at a magnification of × 5400. It did not prove possible to analyse these directly on the QTM 720 because the presence of other organelles of comparable electron density prevented the detection and measurement of the desmosomes alone. In order to overcome this the following technique was adopted. The micrograph of each field was projected onto a sheet of white typing paper at a final magnification of × 36,000 and the cell membrane outlined in green with the areas of specialization—the desmosomes—marked in black. Each drawing of the membrane was then analysed using the QTM 720 epidiascope attachment fitted with the 25 mm lens. Membrane and desmosomes were detected on separate channels and measurements were made of the total projected membrane length, the total length of the desmosomal specializations and their number. From these data we could calculate the percentage of the total projected membrane length occupied by desmosomes, the mean projected desmosome length and the number of desmosomes per unit length of projected membrane (arbitrarily chosen to be 100 cm).

The mean values of the various parameters obtained from each biopsy were then submitted to the Department of Biomathematics, University of Oxford. Dr J. A. Anderson kindly utilized a procedure from the International Computers Limited, 1900 Series Statistical Analysis System Mark 2 (Technical Publication, 4162, 2nd Edn 1969, p. 95). The principle of discriminant analysis is that a *total observation matrix* containing $m$ observations of $n$ variables can be made up into $K$ groups, where $K < m/2$ and where each group contains 2 or more observations. These groups are defined by the user, and subsequently an identification routine can be used to assign a new set of observations to one of these groups, such that the probability of assigning an observation to the wrong group is as small as possible. This analysis thus used all the parameters for each patient to compute a numerical discriminant " score " which, when plotted graphically, allowed direct visual comparison of the relationships between 2 or more groups of patients.

### RESULTS

### 1. *Electron microscopy*

Many of the features which we wished to measure can only be studied satisfactorily with the resolution afforded by the electron microscope. Fig. 1 is an electron micrograph at low power of a cell from the intermediate layer of the ectocervical epithelium, from a patient whose cervix showed no evidence of malignancy. A cell can be seen surrounded by the margins of others, each having multiple processes extending from their surface membrane and making contact with similar processes from the central cell. Desmosomal specializations, visible as dark areas, can be seen at the points of connection. The intercellular space does not react with the contrasting agents used in the preparation of the tissue and hence appears light in the micrograph; this intercellular space we believe to be occupied by mucoprotein complexes which we have partially characterized in a previous publication (Bradbury *et al.*, 1970). The nuclear outline is generally regular and electron-dense nucleoli are often prominent in these nuclei, though not shown in our figure.

FIG. 1.—A low power electron micrograph of a cell from the intermediate zone of a normal human ectocervix. Mitochondria may be seen in the area of cytoplasm adjacent to the nucleus, together with darkly staining bundles of tonofibrils scattered throughout the peripheral cytoplasm. Cytoplasmic projections, terminating in darkly staining desmosomes may be seen crossing the electron-lucent intercellular space. The bar represents 1 $\mu$m.

The ribosomes cannot be identified at this magnification.

Fig. 2 shows a corresponding preparation from a carcinoma of the cervix. Cellular processes appear to be fewer in number than in the normal, and in the majority of instances do not come into contact with the processes from adjacent cells. This lack of cell contact is also associated with an apparent diminution in desmosomal specializations. Visual assessment of the amount of electron-lucent intercellular space suggests no material difference from the normal, an impression not, however, confirmed by measurement (Tables I and II). The nuclei appear to be larger than normal and their outlines are sometimes more indented.

Fig. 3 and 4, taken at higher magnification, show the differences at the sites of cellular contact. In the normal (Fig. 3), the prominent desmosomes can be seen to be associated with a large number of cytoplasmic tonofibrils converging towards them. These are lacking in the carcinoma (Fig. 4). Clusters of ribosomes may be seen in both normal and carcinomatous cells though, once again, visual assessment of their relative numbers is misleading when compared with the actual quantities determined by measurement.

(ii) *Quantitative data.*—Our numerical results are recorded in Fig. 5 and in Tables I and II. The figure shows clearly that the majority of parameters measured show a distinct difference

FIG. 2.—A low power electron micrograph of a cell from a squamous cell carcinoma of the cervix. The cytoplasmic surface projections and desmosomes appear to be reduced in number when compared with the normal cell shown in Fig. 1. Note, however, that the nucleus is appreciably larger and has a more irregular outline. The bar represents 1 μm.

TABLE I.—*Parameters of Normal Cells*

| Code No. | Mean projected nuclear area in μm² | Surface density | No. of ribosomes per μm³ cytoplasm | % Vol. of cytoplasm occupied by tonofibrils | % Vol. of tissue occupied by intercellular space | Desmosomes: % of total projected cell membrane | Desmosomes: Average length (mm) | Desmosomes: No. per 100 cm projected membrane |
|---|---|---|---|---|---|---|---|---|
| 29/70 | 39·1 | 2·5 | 2172 | 21·7 | 42·3 | 7·6 | 13·69 | 4·8 |
| 30/70 | 45·2 | 2·3 | 2708 | 24·2 | 29·3 | 9·0 | 13·58 | 5·6 |
| 35/70 | 41·4 | 2·6 | 2196 | 22·0 | 28·1 | 3·4 | 12·45 | 3·2 |
| 36/70 | 36·0 | 1·9 | 2103 | 12·4 | 28·7 | 8·2 | 12·91 | 5·6 |
| 37/70 | 44·6 | 1·6 | 2081 | 26·0 | 38·5 | 10·6 | 12·53 | 8 |
| 42/70 | 29·3 | 2·2 | 1965 | 23·7 | 32·2 | 7·7 | 10·73 | 4·8 |
| 57/70 | 30·4 | 2·5 | 2377 | 33·4 | 18·7 | 8·9 | 11·12 | 6·4 |
| 64/70 | 43·4 | 3·0 | 2107 | 14·6 | 21·6 | 3·0 | 9 | 3·2 |
| 65/70 | 22·9 | 4·4 | 2790 | 20·9 | 31·0 | 9·0 | 14·17 | 5·6 |
| 72/70 | 43·9 | 2·6 | 2843 | 29·4 | 15·4 | 7·2 | 10·86 | 7·2 |
| 104/71 | 18·6 | 3·0 | 2443 | 23·0 | 22·2 | 8·4 | 11·46 | 6·4 |
| 114/72 | 29·0 | 2·7 | 3025 | 26·2 | 30·9 | 8·7 | 12·37 | 8·8 |
| 134/72 | 55·7 | 2·2 | 2705 | 25·3 | 11·2 | 8·5 | 13 | 6·4 |
| 138/72 | 39·1 | 2·5 | 2105 | 24·4 | 24·2 | 11·5 | 14·82 | 8 |
| 140/72 | 24·4 | 2·3 | 2436 | 24·1 | 40·3 | 6·0 | 7·23 | 9·6 |
| Mean values | 36·2 | 2·55 | 2403·7 | 23·42 | 27·64 | 7·65 | 11·99 | 6·24 |
| s.d. | 10·16 | 0·63 | 335·5 | 5·11 | 8·94 | 2·29 | 2·0 | 1·87 |

Fig. 3.—A high power electron micrograph of the contact regions between the microvillous projections of 2 adjacent cells from the intermediate zone of a normal human ectocervix. Note the lucent intercellular space and numerous tonofibrils in the cytoplasm of each cell converging on the desmosomal specializations which appear as dark thickenings of the cell membrane. Ribosomes are visible in the cytoplasm as minute electron-dense particles. The bar represents 1 $\mu$m.

### Table II.—*Parameters of Carcinomatous Cells*

| Code No. | Mean projected nuclear area in $\mu m^2$ | Surface density | No. of ribosomes per $\mu m^3$ cytoplasm | % vol. of cytoplasm occupied by tonofibrils | % vol. of tissue occupied by intercellular space | Desmosomes: % of total projected cell membrane | Desmosomes: Average length (mm) | Desmosomes: No. per 100 cm of projected membrane | Broders grade |
|---|---|---|---|---|---|---|---|---|---|
| 1/69 | 60·0 | 1·7 | 1887 | 15·4 | 15·4 | 10·5 | 10·78 | 5·6 | II |
| 2/69 | 108·7 | 1·9 | 1862 | 31·1 | 9·8 | 1·1 | 8·61 | 2·4 | III |
| 4/69 | 101·4 | 1·7 | 1050 | 13·2 | 9·8 | 8·8 | 7·69 | 8·8 | III |
| 6/69 | 62·7 | 1·3 | 1735 | 11·4 | 10·9 | 2·5 | 8·41 | 2·4 | III |
| 32B/70 | 105·0 | 2·2 | 2024 | 20·9 | 9·0 | 14·2 | 14·19 | 11·2 | II |
| 52/70 | 49·2 | 2·0 | 1947 | 10·2 | 18·9 | 2·5 | 8·73 | 2·4 | II |
| 54/70 | 62·7 | 2·2 | 1970 | 18·8 | 19·2 | 7·9 | 13·56 | 6·4 | III |
| 71/70 | 112·1 | 2·3 | 2323 | 12·4 | 18·0 | 1·4 | 8·53 | 2·4 | II |
| 79/70 | 84·2 | 2·0 | 2170 | 28·6 | 8·5 | 5·8 | 9·84 | 6·4 | III |
| 80/71 | 112·2 | 2·6 | 2392 | 12·2 | 15·8 | 3·9 | 10·83 | 8·8 | III |
| 81/71B | 95·2 | 2·2 | 1750 | 13·4 | 16·6 | 3·0 | 6·66 | 4 | III |
| 97/71 | 65·0 | 2·0 | 2144 | 19·0 | 12·2 | 8·7 | 12·82 | 5·6 | III |
| 109/71 | 63·9 | 1·4 | 1504 | 11·1 | 7·5 | 4·9 | 10·19 | 6·4 | II |
| 111/71 | 54·0 | 1·2 | 1033 | 16·6 | 12·7 | 13·1 | 11·69 | 11·2 | III |
| 132/72 | 75·7 | 2·4 | 1591 | 20·8 | 25·6 | 3·8 | 7·8 | 5·6 | II |
| 137/72 | 88·5 | 1·7 | 1971 | 17·2 | 7·9 | 4·6 | 8·09 | 6·4 | III |
| Mean values | 81·28 | 1·89 | 1834·56 | 15·89 | 13·61 | 6·04 | 9·9 | 6·0 | |
| s.d. | 22·2 | 0·42 | 390·7 | 4·86 | 5·14 | 4·06 | 2·24 | 2·91 | |

FIG. 4.—A high power electron micrograph of the adjacent border of 2 cancer cells. Two desmosomes only are seen, and there is a reduction in the number of cytoplasmic projections and in the tonofibrils. Ribosomes are visible in the cytoplasm of both cells together with part of the nucleus in the cell at the bottom of the picture. The bar represents 1 $\mu m$.

FIG. 5.—A diagrammatic representation of the distribution and spread of the values obtained in our quantitative analysis of normal and carcinomatous cells.

between the normal and the carcinomatous specimens, though there is a degree of overlap in all cases.

Projected nuclear area appears to give the clearest degree of separation between the populations of normal and carcinomatous cells, although statistical testing of the means (summarized in Table III) by Student's "*t*" test shows

TABLE III.—*Comparison of Mean Values in Normal and Carcinomatous Cells*

| | Student's *t*-test | |
|---|---|---|
| | | *P* |
| Nuclear area | 7·34 | <0·001 (for 29 d.f.) |
| Ribosomes | 4·36 | <0·001 |
| Fibrils | 4·20 | <0·001 |
| Intercellular space | 5·31 | <0·001 |
| Surface density | 3·42 | 0·001 |
| % of cell membrane occupied by desmosomes | 1·51 | <0·2>0·1 |
| No. desmosomes per unit length of membrane | 0·28 | N.S. |
| Average length of desmosomes | 2·74 | 0·01 |

that all the differences are highly significant, with the exception of those for the desmosomes.

From the size-distribution histograms of projected nuclear area, generated by means of the QTM 720 and the Hewlett Packard Calculator, it proved possible to derive a mean nuclear area distribution for both normal and carcinomatous cells. This diagram (Fig. 6) shows clearly the difference between the 2 populations; the normal histogram has a high peak and very little scatter whereas that for the carcinomatous nuclei has a lower peak displaced to the right (*i.e.* the nuclei tend to have a greater area) and shows considerable spread.

Fig. 5 shows that there are generally fewer ribosomes per cubic micrometre of cytoplasm in malignant cells; again it is noticeable that the spread of values for the normal specimens is less than that for the malignant tissues. Analysis of the amount of intercellular space shows that whilst there is less space in malignant

FIG. 6.—A histogram comparing the distribution of nuclear area in normal (N) and carcinomatous (cross hatched, C) cells from the uterine cervix. Note the low peak and wide spread of the values obtained from carcinomatous cells compared with the normal.

tissue than in normal, there is however less spread of the values found in malignant tissue.

The data for both intracytoplasmic tonofibrils and surface density show a similar degree of spread in both populations but those for malignant cells are generally less than for the corresponding normal tissue.

The values obtained for the percentage of cell membrane occupied by desmosomes are very variable in the carcinomatous tissue and, indeed, straddle the complete extent of the normal range (Fig. 5). Superficial examination suggests that there are fewer desmosomes in micrographs of carcinomatous cells, but measurements of their number per unit length (100 cm) of projected cell membrane and calculation of the average length of a desmosome show that no significant difference exists in the number per unit length between normal and abnormal tissue although the average length of the desmosomes in carcinoma appears to be significantly reduced ($P = 0{\cdot}01$).

Comparison of the mean values (Table III) for individual features does not give a full picture of the place of an individual biopsy in the population of either normal or carcinomatous tissues. We have, therefore, included all our data from each biopsy studied in a discriminatory ana-

FIG. 7.—A graphical display of the discriminant scores derived from our raw data. Note that the normal and carcinomatous values fall into 2 completely separate populations.

lysis. This takes into account the relation of each measurement to all others in the same biopsy as well as the relative position of that measurement to all other measurements in the series being investigated. This method of analysis avoids the use of "weighting factors" (as applied by McMaster, 1968) and seems to us to be more objective. As can be seen from Fig. 7, this analysis completely separates patients with classically normal biopsies from those with unquestioned squamous carcinoma.

### DISCUSSION

Bhowmick and Mitra (1968), in a study of the ultrastructural morphology of squamous carcinoma of the human ectocervix, found that there was a great deal of intercellular space and that, unlike normal cells, there were numerous desmosomes between adjacent cell surfaces of the malignant cells. These authors also claim that the intercellular space is decreased to the smallest recorded dimensions in normal epithelium. We have found the opposite with respect to the assessment of both the above parameters. In our material the intercellular space is markedly diminished in the carcinomatous tissue but, although the number of desmosomes is similar in both normal and carcinoma, the desmosomes are shorter in the carcinomatous cells.

Similar studies on the ultrastructure of cervical carcinoma (Hinglais-Guilland *et al.*, 1961) suggested that in the malignant cells there was an increase in the number of microvilli at the cell surface and in the number of desmosomal specializations; again our findings are contrary to those of these authors. It should be noted that the conclusions of both Bhowmick and Mitra, and Hinglais-Guilland *et al.* were based upon subjective estimations and not on a quantitative analysis, which is the basis of this report.

A recent quantitative study is that of Nodskov-Pedersen (1971) who assessed nuclear size of malignant squamous epithelium of the cervix by planimetry. He found that these nuclei were larger than normal but he could not substantiate the work of previous investigators (Atkin and Richards, 1962; Atkin, 1964) who claimed that the nuclear size was related to the prognosis for the patient. We have been unable to show any correlation between nuclear size and the Broders grading of the tumours, the latter being directly related to prognosis (Broders, 1921).

Conclusions based solely upon a single parameter may be inadequate; this was appreciated by McMaster (1968) who concluded that a product should be formed by multiplying together the values of all the parameters from each patient to give a figure, the value of which corresponds with the classification of those particular cervical cells. Such a procedure, however, attaches equal weight to each parameter and this he considered contrary to the practice of cytologists who regard some features, *e.g.* an aberrant chromatin pattern, as of greater significance than an increase in nuclear size. In order to take this into account he devised "weighting factors" which are integral or half-integral indices by which each term is multiplied. The values of these indices were found for each parameter by a computer programme which optimized the total produce to give maximum separation between its value for normal and malignant cells. The justification for these "weighting factors" might be questioned; we agree that several parameters must be assessed but we have not required the addition of "weighting factors", in order to obtain a clear division, into two separate populations, of our measurements of normal and carcinomatous tissues.

Our results for nuclear area and ribosomes show very little overlap between the values for the normal and the malignant cells, the number of ribosomes per cubic micrometre of cytoplasm being smaller than normal in the carcinomatous cells whereas the nuclear area is greater in these latter. Statistical tests show that the difference between the means for these values in normal and malignant tissues is highly significant. It is tempting to suggest that the smaller number of ribosomes in the carcinomatous cell reflects a reduction in protein synthesis. This is supported by the finding of a diminution of the volume of cytoplasmic tonofibrils in these malignant cells. Inspection of our data shows that the overlap in values between the normal and abnormal cells is least for our measurements of nuclear area. This observation is in agreement with the work of Meyer-Arendt and Humphries (1972) who assert that the most characteristic abnormality of cancer cells is the pleomorphism of the cell nuclei and the wide variation in their sizes. Our histogram of projected nuclear area (Fig. 6) shows not only the increase in mean size in carcinomatous nuclei but also the much greater spread of their values.

Although our present data suggest that the enlargement of nuclear size may appear to be the most significant single feature, nevertheless quantitative studies of premalignant stages may reveal that in the early stages more consistent changes may be associated with one of the other parameters. In order to assess this we are currently looking at the relative position of cells obtained from clinical material where the diagnosis ranges from dysplasia to carcinoma *in situ*.

Preliminary studies suggested that there might be a significant decrease in the total number of desmosomal specialization, as reported by McNutt and Weinstein (1969). It is of interest that we have found considerable variation in the number per unit length of membrane, the percentage of membrane occupied by desmosomes and in their average length.

Analysis based on the mean values shows no significant differences between normal and abnormal in the number per unit length of membrane but a low statistical significant ($P = < 0{\cdot}2 > 0{\cdot}1$) for the percentage of cell membrane occupied by the desmosomes. The average length of desmosomes, however, was significantly different, being shorter in the carcinoma ($P = 0{\cdot}01$). The statistical tests were performed on the *mean* values using a parametric test but, in order to ensure that this was justifiable, a cross check using the non-parametric Mann-Whitney $U$ test was used to avoid the assumptions inherent in Student's "$t$" test. The level of significance was similar in both analyses, thus justifying our assumption that the data do not come from populations with the same distribution of values.

Our quantitative analysis has revealed that some of the previously accepted fundamental concepts concerning cancer cells (which were based on visual estimations) are not borne out in the material used in this study. Furthermore, we believe our analysis has shed new light on the relative differences between normal and carcinomatous cells, for example, in the quantity of cytoplasmic tonofibrils and in the number of ribosomes. In our view, at the present time no single parameter should be used on its own but a correlation of at least 5 parameters should be employed to allow a reliable separation between normal and cancerous cells. We hope that extension of this work by other quantitative studies now in progress in our laboratory will provide an earlier and more reliable method of detecting changes that can be shown to be precursors of invasive cervical carcinoma.

We are indebted to our colleagues in the Department of Biomathematics, University of Oxford, Dr J. A. Anderson and Mrs H. J. Amery, for their help with the discriminatory analysis used in the study. Our thanks are due to Dr A. Spriggs for helpful discussion throughout this project. We would like to record the skill and care provided by our Senior technician Mrs Betty Whitehouse and our technicians Miss Carol Nichols, Mrs Marianne Saunders and Miss Valerie Trafford. This study would not have been possible but for the generosity of the Medical Research Council in providing a long-term grant to Dr G. Wiernik and also providing the Quantimet 720 to the Department of Human Anatomy, University of Oxford. The initial stages of preparations were made possible through facilities provided by the Churchill Hospital Research Institute which was built with funds donated by Tenovus, the Oxford Hospital Services Development Trust, the Department of Health and Social Security and the Endowment Funds of the United Oxford Hospitals.

### REFERENCES

ATKIN, N. B. (1964) Nuclear Size in Carcinoma of the Cervix: Its Relation to DNA Content and to Prognosis. *Cancer, N.Y.*, **17**, 1391.

ATKIN, N. B. & RICHARDS, B. M. (1962) Clinical Significance of Ploidy in Carcinoma of the Cervix: Its Relation to Prognosis. *Br. med. J.*, ii, 1445.

BARTELS, P. H., BAHR, G. F., BIBBO, M. & WIED, G. L. (1972) Objective Cell Image Analysis. *J. Histochem. Cytochem.*, **20**, 239.

BHOWMICK, D. K. & MITRA, A. (1968) Ultrastructural Morphology of Squamous Carcinoma of Human Exocervix. *Ind. J. med. Res.*, **56**, 282.

BRADBURY, S., WIERNIK, G., WILLIAMS, E. A. & COWDELL, R. H. (1970) Preliminary Observations on the Histochemistry of the Cell Surface of Carcinoma of the Cervix. *Br. J. Cancer*, **24**, 741.

BRODERS, A. C. (1921) Squamous Cell Epithelioma of the Skin. *Ann. Surg.*, **73**, 141.

DE HOFF, R. T. & RHINES, F. N. (1961) Determination of the Number of Particles per Unit Volume from Measurements made on Random Phase Sections: the General Cylinder and the Ellipsoid. *Trans. AIME*, **221**, 975.

EASSON, E. & RUSSELL, M. H. (1968) (Eds) *The Curability of Cancer in Various Sites.* London: Pitman Medical Publishing Co. Ltd.

FORAKER, A. G. & REAGAN, J. W. (1956) Nuclear Size and Nuclear : Cytoplasmic Ratio in Delineation of Atypical Hyperplasia of Uterine Cervix. *Cancer, N.Y.*, **9**, 470.

FORAKER, A. G. & DENHAM, S. W. (1957) Squamous-cell Carcinoma of Uterine Cervix: Histochemical Review. *Am. J. Obstet. Gynec.*, **74**, 13.

FORAKER, A. G. & REAGAN, J. W. (1959) Nuclear Mass and Allied Phenomena in Normal Exocervical Mucosa, Squamous Metaplasia, Atypical Hyperplasia, Intraepithelial Carcinoma, and Invasive Squamous Cell Carcinoma of the Uterine Cervix. *Cancer, N.Y.*, **12**, 894.

HAGUENAU, F., HOLLMANN, K. H. & ALBOT-PARTURIER, M. (1964) Ultrastructure des cancers du rectum. *Bull. Cancer*, **51**, 55.

HINGLAIS-GUILLAUD, N., MORICARD, R. & BERNHARD, W. (1961) Ultrastructure des Cancers Pavimenteux invasifs du Col Uterin Chez La Femme. *Bull. Cancer*, **48**, 291.

HUSAIN, D. A. N. & HENDERSON, M. J. (1970) Observations on the Use of the Quantimet Image Analysing Computer in Automatic Scanning for Malignant Cells. In *Cytology Automation*. Ed. D. M. D. Evans. Edinburgh: Livingstone.

KAPLAN, B. (1967) Cytomorphological Changes in Intraepithelial Cancer of the Uterine Cervix. *Lav. Anat. pat. Perugia*, **27**, 5.

MCMASTER, G. W. (1968) A Measurement of the Pattern of Normal and Malignant Cervical Cells. *Acta cytologica*, **12**, 9.

MCNUTT, N. S. & WEINSTEIN, R. S. (1969) Ultrastructure of Cell Surfaces in Normal and Neoplastic Cervical Epithelium. *J. cell. Biol.*, **43**, 90a.

MCNUTT, N. S., HERSHBERG, R. A. & WEINSTEIN R. S. (1971) Further Observations on the Occurrence of Nexuses in Benign and Malignant Human Cervical Epithelium. *J. cell Biol.*, **51**, 805.

MEYER-ARENDT, J. R. & HUMPHREYS, D. M. (1972) Quantitative Morphology of Cancer Cells. *Acta histochem.*, **44**, 41.

NODSKOV-PEDERSEN, S. (1971) Degree of Malignancy of Cancer Involving the Cervix Uteri Judged on the Basis of Clinical Stage, Histology, Size of Nuclei and Content of DNA. *Acta path. microbiol. scand.*, Sect. A, **79**, 617.

UNDERWOOD, E. E. (1970) *Quantitative Stereology.* Reading, Mass: Addison-Wesley. *Trans. AIME*, **221**, 975.

WEIBEL, E. R. (1963) *Morphometry of the Human Lung.* Berlin: Springer.

WEIBEL, E. R., KISTLER, G. S. & SCHERLE, W. E. (1966) Practical Stereological Methods for Morphometric Cytology. *J. cell Biol.*, **30**, 23.

WEIBEL, E. R. & ELIAS, H. (1967) *Quantitative Methods in Morphology.* New York: Springer.

WEIBEL, E. R. (1969) Stereological Principles for Morphometry in Electron Microscopic Cytology. *Int. rev. Cytol.*, **26**, 235.

YOUNES, M. S. (1969) Electron Microscope Observations on Carcinoma *In Situ* of the Cervix. *Obstet. Gynec. Surv.*, **24**, 768.